# Optimization of fiber reinforcement in hand-pour epoxy composites for developing low-cost canine composite bone models: Mechanical characterization and material selection

**DOI:** 10.14202/vetworld.2026.3191-3204

**Published:** 2026-07-20

**Authors:** Somchai Sompaisarnsilp, Suwaree Vosbein, Athicom Chin-on, Nattapon Chantarapanich

**Affiliations:** 1 Faculty of Veterinary Medicine, Rajamangala University of Technology Tawan-ok, Chonburi 20110, Thailand.; 2 Department of Mechanical Engineering, Faculty of Engineering at Sriracha, Kasetsart University. Sriracha, Chonburi 20230, Thailand.

**Keywords:** canine bone model, epoxy resin, fiber reinforcement, hand-pour casting, mechanical characterization, orthopedic training, polyurethane foam, veterinary education

## Abstract

**Background and Aim:**

Synthetic canine bone models are increasingly used in veterinary orthopedic education as reproducible and ethically acceptable alternatives to cadaveric specimens. However, commercially available models remain expensive, and low-cost fabrication methods that provide suitable mechanical performance are limited. This study aimed to optimize fiber-reinforced epoxy resin (E) and polyester resin (R) composites fabricated by a hand-pour casting technique for the development of affordable canine composite bone models by comparing their tensile mechanical properties and identifying the most suitable cortical bone analog material.

**Materials and Methods:**

E and R were reinforced with chopped E-glass fiber at 0%, 1%, 3%, and 5% (wt/wt), whereas polyurethane (PU) foam was evaluated as a cancellous bone analog. Dumbbell-shaped specimens were fabricated using silicone molds according to American Society for Testing and Materials D638-14 and tested under uniaxial tension using a universal testing machine. Elastic modulus, yield strength, yield strain, ultimate stress, and ultimate strain were determined from stress–strain curves. Mechanical properties were compared using one-way analysis of variance followed by Tukey’s honestly significant difference test, with statistical significance set at p < 0.05.

**Results:**

Fiber reinforcement significantly improved the elastic modulus of epoxy composites in a concentration-dependent manner, reaching 1,513.12 ± 67.85 MPa at 5 wt%, whereas polyester composites showed no significant stiffness improvement. Epoxy composites demonstrated markedly lower specimen-to-specimen variability than polyester composites, with coefficients of variation for elastic modulus ranging from 1.4% to 4.5%. Although ultimate stress and ultimate strain decreased with increasing fiber content in both resin systems, epoxy reinforced with 3 wt% E-glass fiber provided the most favorable balance between stiffness, reproducibility, and casting workability. PU foam exhibited an elastic modulus of 57.72 ± 7.69 MPa, providing a 23-fold stiffness difference from the selected cortical analog and supporting its suitability as a cancellous core material. The estimated raw material cost of the proposed composite bone model was approximately 7% of the equivalent commercial product.

**Conclusion:**

E reinforced with 3 wt% chopped E-glass fiber is the preferred material for low-cost hand-pour fabrication of canine composite bone models because it offers the optimal combination of mechanical performance, manufacturing consistency, and processing feasibility. Combined with PU foam, this formulation provides an economical platform for veterinary surgical simulation. Future studies should validate the bilayer construct under compressive loading, drilling, and screw pull-out testing before clinical educational application.

## INTRODUCTION

Experiential fracture stabilization training is a cornerstone of veterinary surgical education. Cadaveric bone, the traditional training substrate, has several well-recognized limitations, including anatomical variability, the potential risk of disease transmission, chemical hazards associated with preservation, and high procurement and maintenance costs [1–4]. Consequently, synthetic canine composite bone models (CBMs) have gained increasing acceptance as supplementary educational tools because they provide reproducibility, improve animal welfare by reducing cadaver use, and offer greater logistical convenience [5, 6]. To function as effective surgical surrogates, CBMs should reproduce the hierarchical mechanical architecture of natural canine bone, comprising a dense cortical shell with a stiffness of 7.5–21 GPa and a tensile strength of approximately 251 MPa [7], surrounding a porous trabecular core optimized for compressive load absorption [8]. Epoxy resin (E), polyester resin (R), and polyurethane (PU) foam are commonly used to fabricate such models because of their low-cost, ease of processing, and versatility. However, these materials exhibit inherent brittleness and do not fully replicate the mechanical behavior of native bone, thereby limiting their effectiveness for advanced orthopedic procedural training [9–14].

Fiber reinforcement has been widely employed to improve the stiffness and fracture toughness of polymer composites while maintaining relatively low production costs [15–19]. Nevertheless, increasing fiber content also increases resin viscosity, making it difficult to cast composites into the small and geometrically complex molds required for canine bone models. Compared with human bone analogs, canine-specific molds require tighter dimensional tolerances within substantially smaller casting volumes, and the mechanical behavior of fiber-reinforced composites under these manufacturing conditions cannot be directly extrapolated from studies of human bone models [20, 21]. Therefore, identifying a fiber weight fraction that simultaneously provides adequate resin workability and desirable biomimetic mechanical properties remains a major challenge for developing canine-specific CBM.

Veterinary institutions across the Association of Southeast Asian Nations (ASEAN) are progressively aligning their curricula with international Day-1 Competency standards, resulting in an increasing demand for simulation-based surgical training [22, 23]. However, commercially available bone simulators remain prohibitively expensive for many veterinary schools, particularly in resource-limited settings. Hand-pour casting using locally available E or R together with PU foam represents a practical and economical fabrication approach. PU foam is particularly suitable as a cancellous bone analog because of its adjustable density, established application in orthopedic simulation, and widespread commercial availability in Thailand [13, 14]. Previous studies have shown that fiber reinforcement can improve the stiffness and fracture resistance of open-cast polymer composites within the 0.5–5 wt% range; however, increasing fiber content progressively compromises resin flowability, mold filling, and structural integrity [15, 18, 24]. The optimal balance between mechanical enhancement and manufacturing feasibility for canine-sized CBM has not yet been established.

Although previous investigations have evaluated fiber-reinforced polymer composites and commercially available synthetic bone models, most have focused on human orthopedic applications or general composite material characterization rather than canine-specific surgical simulation [9, 10, 15–21]. Furthermore, existing studies have largely emphasized improvements in mechanical properties without simultaneously considering casting workability, specimen reproducibility, and cost-effectiveness under hand-pour fabrication conditions. As a result, there remains insufficient evidence regarding the optimal fiber weight fraction for E- and R-based composites intended for low-cost canine CBMs that can reproduce the mechanical hierarchy of cortical and cancellous bone while remaining practical for routine fabrication in veterinary teaching institutions.

Therefore, this study aimed to optimize hand-pour fabricated fiber-reinforced E and R composites for developing affordable canine CBMs by systematically evaluating fiber weight fractions of 1, 3, and 5 wt%. The tensile mechanical properties of the reinforced resin systems were compared with those of PU foam as a cancellous bone analog and with published mechanical properties of natural canine cortical and trabecular bone. The findings were used to identify the composite formulation that provides the most appropriate balance between mechanical performance, manufacturing reproducibility, casting feasibility, and economic affordability for veterinary orthopedic training. To the best of our knowledge, this is the first study to systematically optimize fiber weight fractions in E and R composites specifically for hand-pour fabrication of canine orthopedic training models.

## MATERIALS AND METHODS

### Ethical approval

This study did not involve live animals, animal tissues, or biological specimens and therefore did not require approval from an Institutional Animal Care and Use Committee or any other institutional ethics committee. The study was conducted exclusively on synthetic materials for the development of canine CBMs. The research was undertaken in accordance with the principles of the 3Rs (Replacement, Reduction, and Refinement), with the objective of reducing the use of cadaveric specimens in veterinary surgical education by developing a reproducible and cost-effective synthetic training model.

### Study period and location

The study was conducted from November 2021 to May 2022 at the Faculty of Veterinary Medicine, Rajamangala University of Technology Tawan-ok, Chonburi, Thailand. Mechanical testing was performed at the Department of Mechanical Engineering, Faculty of Engineering, Kasetsart University Sriracha Campus, Chonburi, Thailand.

### Study design

This experimental materials engineering study was designed to optimize the mechanical performance of hand-pour fabricated fiber-reinforced polymer composites for canine CBMs. R and E were evaluated as cortical bone analog materials, whereas PU foam was evaluated as a cancellous bone analog. Four fiber reinforcement levels (0, 1, 3, and 5 wt%) were investigated for each resin system. Tensile mechanical properties were determined according to American Society for Testing and Materials (ASTM) D638-14 and compared to identify the formulation providing the best balance between mechanical performance, manufacturing reproducibility, and casting feasibility.

### Specimens

R and E were selected as candidate materials for the cortical bone analog, whereas PU foam was selected as the cancellous bone analog. R (R804) was obtained from Super Silicone & Resin Art, Bangkok, Thailand. E (EP-089), E-glass fiber filament, and PU foam were obtained from Concrete Composite Co., Ltd., Bangkok, Thailand. The principal properties of the polyester and Es provided by the manufacturers are summarized in Table 1.

**Table 1:** Comparison of key properties of the polyester and epoxy resins used in this study.

**Properties**	**Polyester resin R804**	**Epoxy resin EP-089**
Appearance	Turbid pink liquid	Clear transparent viscous liquid
Viscosity (cP at 25°C)	300–400	11,500–15,000
Density (g/cm³ at 25°C)	NA	1.16
Total solid content (%)	56 ± 2	NA
Gel time (min)	15–25	30
Curing time (h)	1–2	4–6
Appearance after curing	Pink solid	Nearly transparent solid
Cost (USD/kg)	7.56	19.01

**Figure 1:**
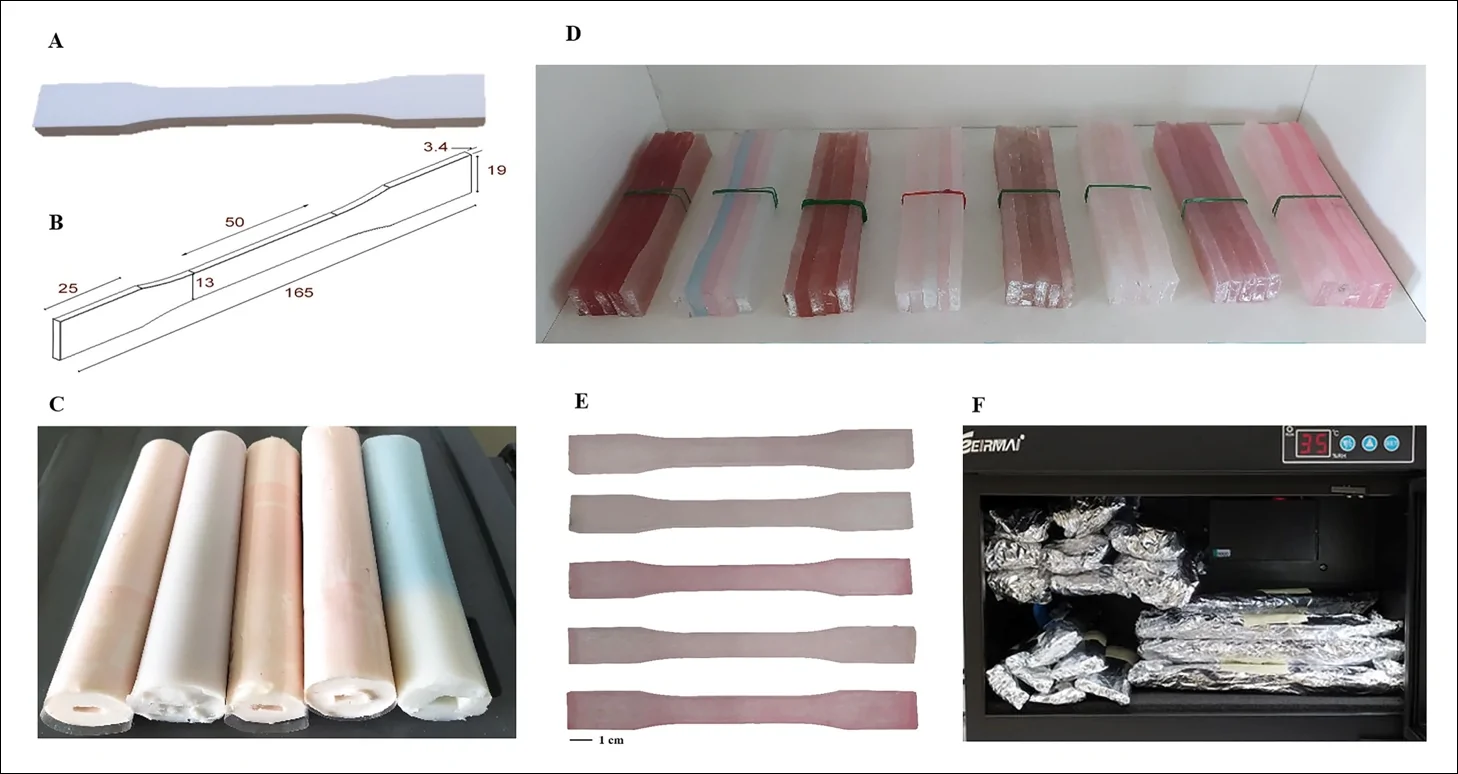
Preparation and characterization of tensile test specimens. (A) Template for mold fabrication. (B) Standard specimen dimensions according to ASTM D638-14 (mm). (C) Silicone mold. (D) Dumbbell-shaped tensile specimens prepared using the custom mold. From left to right: R+1%RF, E+1%RF, R+3%RF, E+3%RF, R+5%RF, E+5%RF, R, and E. R = polyester resin; E = epoxy resin; RF = fiber reinforcement weight fraction. Scale is shown in (E). (E) Top-view photograph of tensile specimens (scale bar = 1 cm). (F) Specimens stored in a digitally controlled dehumidifying cabinet.

Five specimens were fabricated for each material composition using a custom silicone rubber mold (Figure 1C) at room temperature (25°C) according to ASTM D638-14 Type I specifications [25] (Figure 1A and B). For the cortical bone analog, chopped 6-mm E-glass fibers were incorporated into the resin matrix at fiber reinforcement levels of 0, 1, 3, and 5 wt%.

For E (EP-089), Parts A and B were mixed at a volumetric ratio of 2:1. For R (R804), catalyst was added at 1.5% (w/w). From the second mention onward, Super Silicone & Resin Art and Concrete Composite Co., Ltd. are referred to by company name only.

For fiber-reinforced specimens, chopped fibers were gradually incorporated into the base resin before addition of the hardener or catalyst while continuously stirring with a flat spatula to ensure complete wetting of each fiber increment. After reaching the desired fiber weight fraction, the hardener (E) or catalyst (R) was added, and the mixture was stirred until visually homogeneous (approximately 1–2 min).

To minimize air entrapment, mixing was performed using a continuous figure-of-eight motion while maintaining the spatula below the liquid surface. The resin mixture was subsequently poured into paste wax-treated molds from a height of approximately 30 cm using a thin continuous stream to facilitate bubble release. Vacuum degassing was not performed. Fiber distribution within the cured specimens was verified by visual examination of the fracture cross-section after tensile testing. Initial curing before demolding required approximately 2 h for R and 8 h for E. Specimens exhibiting visible voids, trapped air bubbles, or surface defects were excluded from subsequent analyses.

For the cancellous bone analog, a two-component PU foam consisting of polyol (Part A) and isocyanate (Part B) was prepared at a 1:1 volumetric ratio according to the manufacturer's instructions. The mixture was stirred for approximately 30 s until homogeneous. The system expanded to approximately 25 times its original liquid volume, producing a low-density closed-cell foam with a free-rise density of 27–31 kg/m³. Following casting, the foam reached its maximum expansion within approximately 1 min.

The apparent density of each cured PU specimen was determined gravimetrically by dividing specimen mass (measured to 0.01 g) by specimen volume calculated from ASTM D638-14 Type I dimensions using digital caliper measurements of specimen length, width, and thickness (Figure 1B). Following demolding, all specimens were conditioned for at least 48 h in a digitally controlled dehumidifying cabinet maintained at 23 ± 2°C and 50 ± 5% relative humidity before mechanical testing.

### Mechanical testing

**Figure 2:**
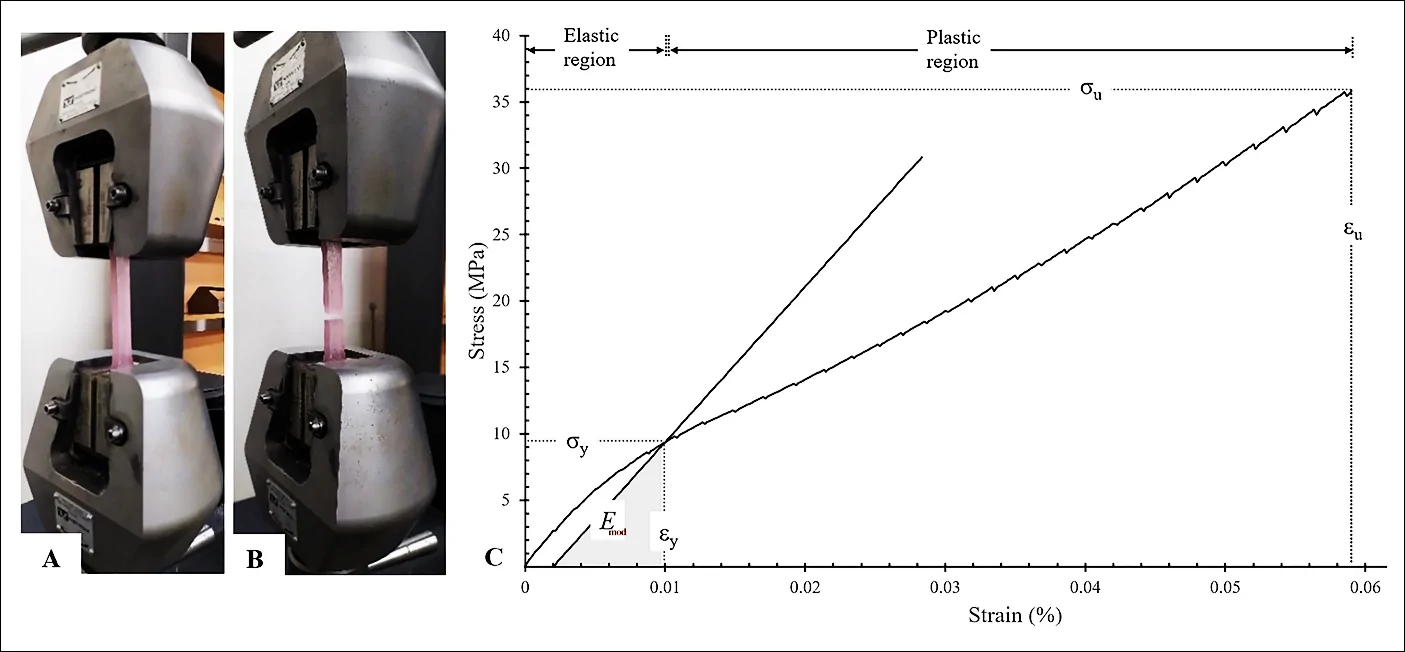
Tensile testing procedure. (A) Specimen mounted before testing. (B) Specimen after tensile failure. (C) Representative stress-strain curve showing the linear regression (dashed line) fitted to the initial elastic region. *Emod* = elastic modulus; σy = yield strength; εy = yield strain; σu = ultimate stress; εu = ultimate strain.

All specimens were subjected to uniaxial tensile testing at 25°C using a universal testing machine (Instron 5982; Instron Inc., Norwood, MA, USA) equipped with a 100-kN load cell and self-tightening wedge grips. Dumbbell-shaped specimens prepared according to ASTM D638 Type I with a 50-mm gauge length (Figures 1D and E) were tested at a constant crosshead speed of 5.0 mm/min. Force and crosshead displacement were continuously recorded at a sampling frequency of 10 Hz until specimen failure (Figures 2A and B).

Engineering strain (ε) was calculated from crosshead displacement as:


\[
\varepsilon =\frac{\Delta L}{{L}_{0}}
\]

where ΔL is the change in gauge length after testing and L₀ is the original gauge length.

Because strain measurements were derived from crosshead displacement rather than a contact or non-contact extensometer, the calculated strain values may slightly overestimate absolute strain because of machine compliance and grip slippage. Nevertheless, all specimens were tested under identical experimental conditions, allowing reliable relative comparisons among groups. Therefore, strain-related parameters (εy and εu) should be interpreted comparatively rather than as absolute material constants.

Engineering tensile stress (σ) was calculated as:


\[
\sigma =\frac{P}{{A}_{0}}
\]

where *P* is the applied load and *A₀* is the original cross-sectional area, determined from the mean of three digital micrometer measurements obtained at the center of the gauge section.

Stress–strain curves were generated for all specimens. The elastic modulus (*Emod*) was calculated from linear regression of the maximum slope within the linear elastic region when R² exceeded 0.90. Yield strength (σy) and yield strain (εy) were determined using the 0.2% offset method [26, 27]. Ultimate stress (σu) and ultimate strain (εu) were recorded for each specimen (Figure 2C).

### Statistical analysis

A minimum of five specimens per group was selected according to ASTM D638 recommendations for material characterization studies [26] and was also limited by the fabrication capacity of each molding batch. The unreinforced E group contained six specimens because one additional specimen met all predefined quality criteria and was therefore included in the analysis.

Mean ± standard deviation were calculated for *Emod*, σy, εy, σu, and εu. Data normality was evaluated using the Shapiro–Wilk test, whereas homogeneity of variance was assessed using Levene's test. Differences among groups were analyzed using one-way analysis of variance (ANOVA), followed by Tukey's honestly significant difference (HSD) test for multiple comparisons when appropriate. Cohen's *d* was calculated to estimate effect sizes for statistically significant pairwise comparisons.

Mechanical properties between the E+3%RF and PU groups were additionally compared using Welch's independent-samples *t*-test. All statistical tests were two-sided, and statistical significance was established at p < 0.05. Statistical analyses were performed using R statistical software version 4.5.3 [28].

## RESULTS

### Overall tensile mechanical properties

**Figure 3:**
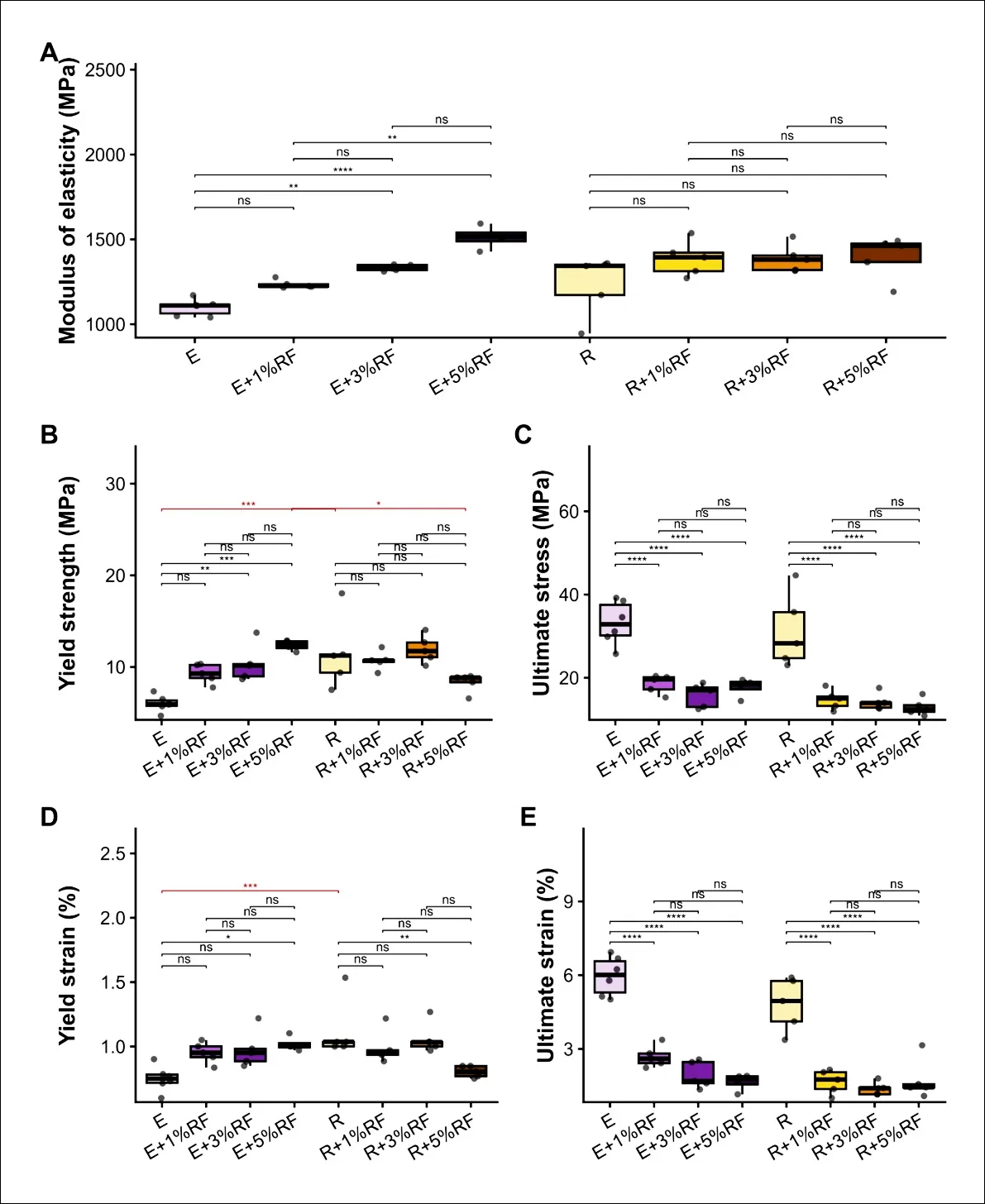
Tensile mechanical properties of epoxy and polyester resin composites across fiber reinforcement concentrations. (A) Elastic modulus. (B) Yield strength. (C) Ultimate stress. (D) Yield strain. (E) Ultimate strain for each specimen group. ns = p ≥ 0.05; * = p < 0.05; ** = p < 0.01; *** = p < 0.001.

The mechanical properties of all specimen groups determined by uniaxial tensile testing are summarized in Table 2. One-way ANOVA revealed significant between-group differences in all five mechanical parameters: *Emod* (F(7,32) = 9.253, p < 0.001), yield strength (σy) (F(7,32) = 7.198, p < 0.001), yield strain (εy) (F(7,32) = 5.200, p < 0.001), ultimate stress (σu) (F(7,32) = 18.635, p < 0.001), and ultimate strain (εu) (F(7,32) = 36.411, p < 0.001). Normality of residuals was violated for four of the five parameters (Shapiro–Wilk test, p < 0.05), and homogeneity of variance was violated for three of the five parameters (Levene's test, p < 0.05); however, one-way ANOVA was retained for all parameters because it is considered robust to moderate violations of these assumptions when group sizes are approximately equal (Supplementary Table S1). The results of post hoc pairwise comparisons using Tukey's HSD test are presented in Figure 3. The coefficient of variation for *Emod* was consistently lower in the epoxy groups (1.4%–4.5%) than in the polyester groups (5.9%–14.5%), and this pattern persisted across both unreinforced and all fiber-reinforced groups (Table 2).

### Effect of fiber reinforcement on E and R composites

In the absence of fiber reinforcement, R exhibited significantly higher yield strength (11.51 ± 3.97 MPa; d = 2.011, p < 0.001) and yield strain (1.12 ± 0.23%; d = 2.171, p < 0.001) than E (6.02 ± 0.88 MPa and 0.75 ± 0.10%, respectively). However, *Emod*, ultimate stress (σu), and ultimate strain (εu) did not differ significantly between the two unreinforced resins (p > 0.05 for all). The maximum fiber weight fraction at which hand-pour casting into the silicone mold remained feasible was 5 wt%.

**Table 2:** Mean ± SD mechanical properties of all specimen groups determined by uniaxial tensile testing.

**No.**	**Group**	**n**	***Emod*** ** (MPa)**	**σy** ** (MPa)**	**εy** ** (%)**	**σu** ** (MPa)**	**εu** ** (%)**	**Coefficient of variation for ** ***Emod*** ** (%)**
1	E	6†	1,099.52 ± 48.62	6.02 ± 0.88	0.75 ± 0.10	33.17 ± 5.24	5.96 ± 0.79	4.4
2	E+1%RF	5	1,234.84 ± 24.43	9.28 ± 1.07	0.95 ± 0.08	18.52 ± 2.22	2.69 ± 0.44	2.0
3	E+3%RF	5	1,333.06 ± 18.42	10.38 ± 2.02	0.98 ± 0.14	15.78 ± 2.87	1.93 ± 0.55	1.4
4	E+5%RF	4‡	1,513.12 ± 67.85	12.37 ± 0.59	1.02 ± 0.06	17.71 ± 2.27	1.66 ± 0.35	4.5
5	R	5	1,234.28 ± 179.32	11.51 ± 3.97	1.12 ± 0.23	31.28 ± 8.92	4.82 ± 1.08	14.5
6	R+1%RF	5	1,387.52 ± 103.47	10.71 ± 1.00	0.99 ± 0.13	14.75 ± 2.36	1.67 ± 0.48	7.5
7	R+3%RF	5	1,387.24 ± 81.92	11.93 ± 1.50	1.06 ± 0.12	14.19 ± 2.00	1.39 ± 0.27	5.9
8	R+5%RF	5	1,397.44 ± 125.41	8.32 ± 1.02	0.80 ± 0.04	12.89 ± 2.00	1.73 ± 0.81	9.0
9	PU	5	57.72 ± 7.69	1.14 ± 0.25	2.17 ± 0.24	1.76 ± 0.38	4.04 ± 1.26	13.3

Note: All values are presented as mean ± SD. Strain values are reported as percentages (%). *Emod* = elastic modulus; σy = yield strength; εy = yield strain; σu = ultimate stress; εu = ultimate strain; R = polyester resin; E = epoxy resin; RF = fiber reinforcement weight fraction; PU = polyurethane foam. †n = 6 for the epoxy resin control group; all other groups had n = 5 unless otherwise indicated. ‡n = 4; one specimen was excluded because of mechanical failure during gripping before testing.

**When reinforced, the Emod of E increased with increasing fiber weight fraction:** E+1%RF (1,234.84 ± 24.43 MPa), E+3%RF (1,333.06 ± 18.42 MPa), and E+5%RF (1,513.12 ± 67.85 MPa). Significant differences from unreinforced E (1,099.52 ± 48.62 MPa) were detected at 3 wt% (d = −6.103, p < 0.01) and 5 wt% (d = −7.307, p < 0.001), representing a 37.6% increase at the highest concentration. The difference at 1 wt% did not reach statistical significance (p > 0.05). No significant change in *Emod* was observed across any fiber weight fraction in R (p > 0.05 for all pairwise comparisons; Figure 3A; Supplementary Table S2).

Yield strength increased significantly in epoxy composites at 3 wt% (10.38 ± 2.02 MPa; d = −2.914, p < 0.01) and 5 wt% (12.37 ± 0.59 MPa; d = −8.076, p < 0.001) relative to unreinforced E (6.02 ± 0.88 MPa); however, the difference at 1 wt% (9.28 ± 1.07 MPa) was not statistically significant (p > 0.05). In polyester composites, no significant pairwise difference in yield strength was detected among any of the four fiber weight fractions (p > 0.05 for all). E+5%RF exhibited significantly higher yield strength than R+5%RF (12.37 ± 0.59 MPa vs. 8.32 ± 1.02 MPa; d = 4.710, p < 0.05; Figure 3B).

Ultimate stress decreased significantly with fiber addition in both resin systems relative to their respective unreinforced controls (Figure 3C). For epoxy composites, significant reductions from unreinforced E (33.17 ± 5.24 MPa) were observed at 1 wt% (18.52 ± 2.22 MPa; d = 3.505, p < 0.001), 3 wt% (15.78 ± 2.87 MPa; d = 3.996, p < 0.001), and 5 wt% (17.71 ± 2.27 MPa; d = 3.534, p < 0.001). For polyester composites, significant reductions from unreinforced R (31.28 ± 8.92 MPa) were observed at 1 wt% (14.75 ± 2.36 MPa; d = 2.535, p < 0.001), 3 wt% (14.19 ± 2.00 MPa; d = 2.645, p < 0.001), and 5 wt% (12.89 ± 2.00 MPa; d = 2.847, p < 0.001).

Among the five mechanical parameters, yield strain (εy) showed the least sensitivity to fiber reinforcement. In epoxy composites, only E+5%RF (1.02 ± 0.06%) differed significantly from unreinforced E (0.75 ± 0.10%; d = −3.129, p < 0.05); differences at 1 wt% and 3 wt% were not significant (p > 0.05). In polyester composites, only R+5%RF (0.80 ± 0.04%) differed significantly from unreinforced R (1.12 ± 0.23%; d = 1.903, p < 0.01); differences at 1 wt% and 3 wt% were not significant (p > 0.05; Figure 3D).

Ultimate strain decreased significantly with fiber addition at all three concentrations in both resin systems (Figure 3E). For epoxy composites, reductions from unreinforced E (5.96 ± 0.79%) were significant at 1 wt% (2.69 ± 0.44%; d = 4.960, p < 0.001), 3 wt% (1.93 ± 0.55%; d = 5.790, p < 0.001), and 5 wt% (1.66 ± 0.35%; d = 6.488, p < 0.001). For polyester composites, reductions from unreinforced R (4.82 ± 1.08%) were significant at 1 wt% (1.67 ± 0.48%; d = 3.771, p < 0.001), 3 wt% (1.39 ± 0.27%; d = 4.363, p < 0.001), and 5 wt% (1.73 ± 0.81%; d = 3.230, p < 0.001).

### Stress–strain behavior of the resin composites

**Figure 4:**
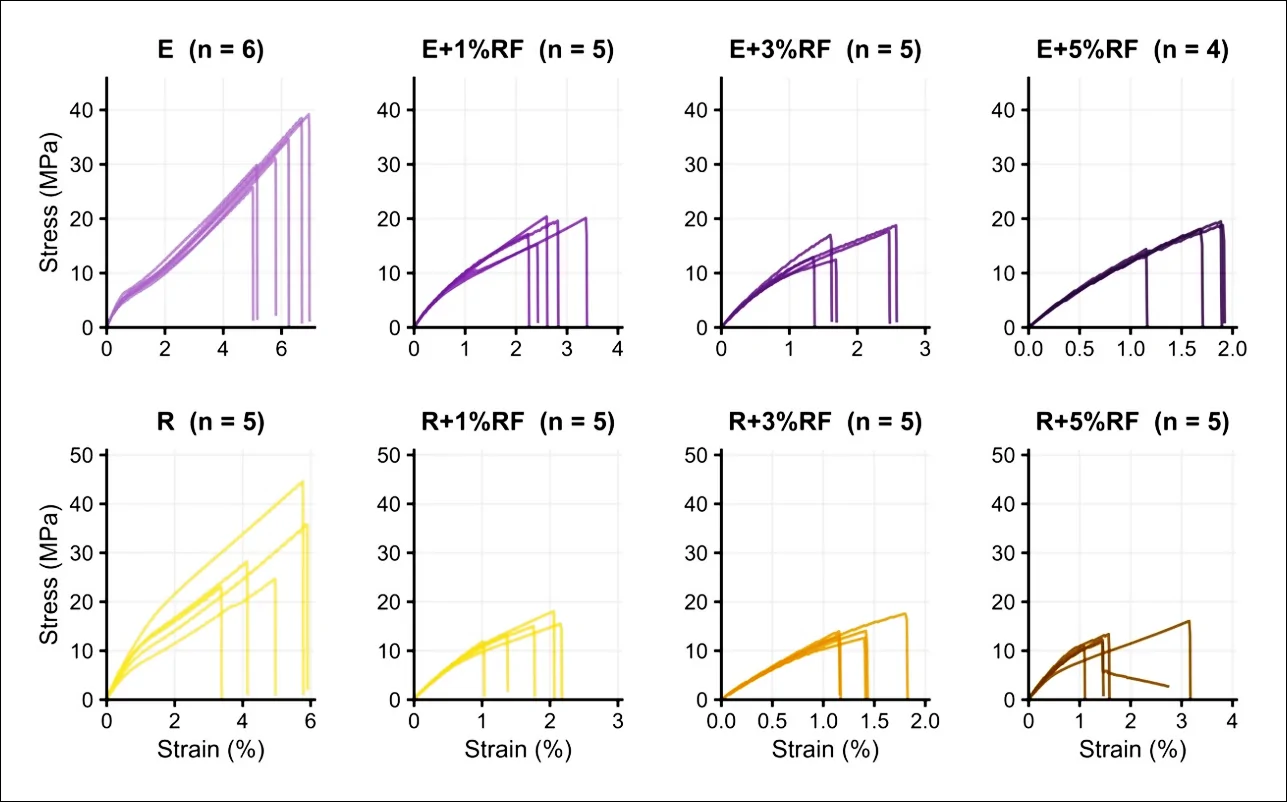
Stress–strain curves of individual specimens. E = epoxy resin; R = polyester resin; RF = fiber reinforcement weight fraction. The x-axis scales differ between panels to optimize the readability of the individual curve morphology. Absolute strain values for each group are presented in Table 2.

The stress-strain curves for all specimen groups are shown in Figure 4. Curves for all groups exhibited an initial toe region followed by a linear elastic region and abrupt brittle failure. Unreinforced E specimens displayed a two-slope response, characterized by a steeper initial elastic region transitioning to a reduced-slope region before failure, which is consistent with the 0.2% offset method used for yield determination. Unreinforced R specimens showed a more continuously curvilinear ascent with greater inter-specimen variation (coefficient of variation for *Emod* = 14.5%; Table 2). With increasing fiber weight fraction in both resin systems, the post-toe response became progressively steeper and more linear, with correspondingly more abrupt failure. All fiber-reinforced specimens failed within the gauge section by abrupt brittle fracture (representative failure shown in Figure 2B), whereas unreinforced epoxy and polyester specimens exhibited visible localized deformation at the fracture plane before failure.

### Mechanical comparison between E+3%RF and PU foam

**Figure 5:**
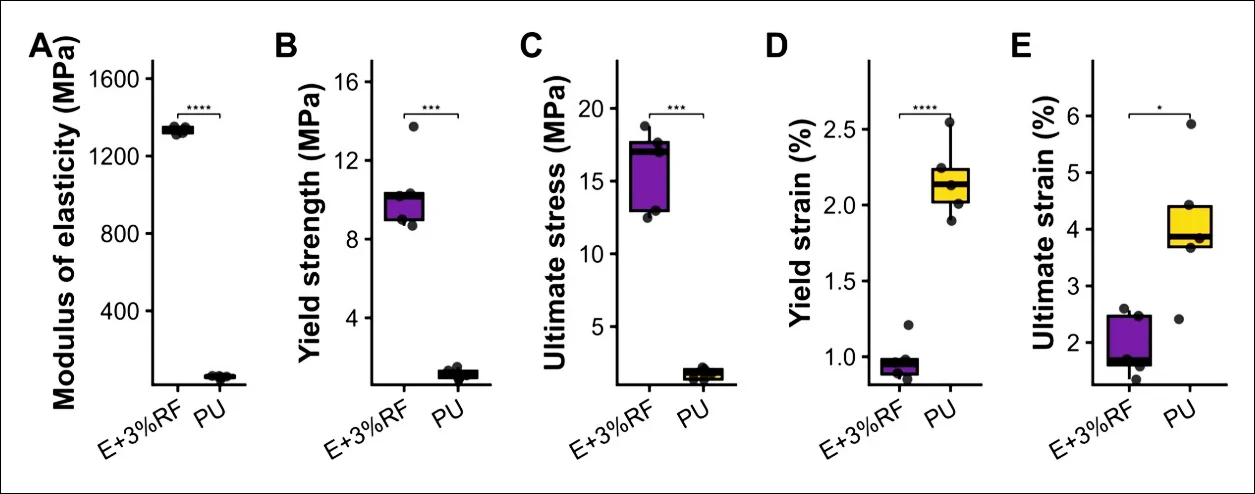
Mechanical property comparison between E+3%RF and PU foam. (A) Elastic modulus. (B) Yield strength. (C) Ultimate stress. (D) Yield strain. (E) Ultimate strain. ns = p ≥ 0.05; * = p < 0.05; *** = p < 0.001; **** = p < 0.0001.

The mold-constrained apparent density of the PU foam specimens was 280 ± 4 kg/m³ (mean ± SD; n = 5). Welch's independent-samples *t*-test detected significant differences between PU foam and E+3%RF across all five mechanical parameters (Figure 5). Elastic modulus (*Emod*) (57.72 ± 7.69 MPa; t(5.35) = 142.891, d = 90.372, p < 0.001), yield strength (σy) (1.14 ± 0.25 MPa; t(4.12) = 10.174, d = 6.435, p < 0.001), and ultimate stress (σu) (1.76 ± 0.38 MPa; t(4.14) = 10.817, d = 6.841, p < 0.001) were all substantially lower in PU foam than in E+3%RF (1,333.06 ± 18.42 MPa, 10.38 ± 2.02 MPa, and 15.78 ± 2.87 MPa, respectively). Yield strain (εy) (2.17 ± 0.24%; t(6.55) = −9.436, d = −5.968, p < 0.001) and ultimate strain (εu) (4.04 ± 1.26%; t(5.46) = −3.427, d = −2.167, p < 0.05) were significantly higher in PU foam than in E+3%RF (0.98 ± 0.14% and 1.93 ± 0.55%, respectively).

## DISCUSSION

### Mechanical performance of the cortical bone analogs

The primary mechanical benchmark for a cortical bone analog is the elastic modulus of natural canine cortical bone, reported across skeletal sites and breeds in the range of approximately 7.5–21.0 GPa [7]. The highest modulus achieved in the present study (E+5%RF: 1,513.12 ± 67.85 MPa) represents approximately 20% of the lower bound of that target range. This shortfall is consistent with the known stiffness ceiling of short-fiber thermoset composites fabricated by manual open-cast molding, in which fiber length distribution, random orientation, and residual porosity limit load transfer efficiency [30, 31, 32]. No equivalent modulus response was observed in the polyester system, in which elastic modulus remained statistically unchanged across all three weight fractions (p > 0.05 for all pairwise comparisons), a finding that distinguishes the two resin matrices and has practical implications for formulation selection in low-resource fabrication contexts.

This difference between the two resin systems is most likely attributable to cure shrinkage. Unsaturated R undergoes volumetric shrinkage of 7%–12% during polymerization, compared with <2% for E [36]. In an open-cast, unpressurized format, as used in the present study, this shrinkage generates residual stresses at the fiber-matrix interface, promotes micro-debonding, and reduces the efficiency of load transfer from the matrix to the fiber [37, 38]. The result is that fiber addition in R produced no statistically detectable stiffness gain at any weight fraction tested. Manufacturing consistency followed the same pattern: the coefficient of variation for elastic modulus was 1.4%–4.5% across the epoxy groups and 5.9%–14.5% across the polyester groups at equivalent fiber weight fractions (Table 2). A coefficient of variation of 14.5% means that specimens of nominally identical composition will behave differently during surgical training, which defeats the purpose of a standardized synthetic model. On both counts—stiffness response to reinforcement and specimen-to-specimen consistency—E is the preferable matrix for hand-pour canine bone model fabrication.

### Effect of fiber reinforcement on tensile properties

Fiber reinforcement reduced ultimate tensile stress and ultimate strain in both resin systems despite increasing elastic modulus. In E, ultimate stress fell from 33.17 ± 5.24 MPa to 17.71 ± 2.27 MPa at 5 wt%, representing a 46.6% reduction, and ultimate strain decreased from 5.96 ± 0.79% to 1.66 ± 0.35%. R showed a comparable decline, with ultimate stress decreasing from 31.28 ± 8.92 MPa to 12.89 ± 2.00 MPa and ultimate strain decreasing from 4.82 ± 1.08% to 1.73 ± 0.81%, respectively (Table 2). This finding is consistent with fiber agglomeration and void formation at higher weight fractions in hand-mixed systems, generating stress concentrators that initiate premature matrix failure under tensile loading [39, 40]. The reduction in ductility does not, however, necessarily impair training utility. Canine cortical bone has an ultimate tensile strain of approximately 1%–3% [30, 41], and unreinforced E at 5.96% already exceeds this range substantially; epoxy composites containing 3–5 wt% fiber reinforcement (1.93%–1.66%) fall within or immediately below this range, representing a closer approximation to natural tissue. Whether the reduced ultimate stress at higher fiber weight fractions causes premature or inconsistent fracture under drilling and sawing loads cannot be determined from tensile coupon data alone.

### Selection of the optimal cortical bone formulation

The maximum fiber weight fraction at which hand-pour casting into the silicone mold remained feasible was 5 wt%; beyond this concentration, the increasing viscosity of the fiber-laden mixture precluded complete mold filling under the manual open-cast conditions used in the present study. Within the range examined, E+3%RF is the recommended formulation for reproducible hand-pour canine bone model fabrication. Although E+5%RF achieved a higher elastic modulus (1,513.12 ± 67.85 MPa vs. 1,333.06 ± 18.42 MPa), reproducibility of that property across fabricated specimens is a practical requirement for standardized surgical training models [10]. On this criterion, E+3%RF is superior because its coefficient of variation for elastic modulus was 1.4%, the lowest recorded across all nine groups, compared with 4.5% for E+5%RF. The reliability of the E+5%RF estimate is further reduced by the loss of one specimen because of pre-test grip failure, yielding n = 4, the smallest group in the study, which limits confidence in both the mean value and the coefficient of variation at that concentration. Both E+3%RF and E+5%RF reached only 17.8% and 20.2%, respectively, of the lower bound of the canine cortical bone target (7,500 MPa); therefore, a 2.4 percentage-point difference at this scale of approximation does not constitute a meaningful biomimetic advantage for either formulation. E+3%RF therefore offers the best available balance of mechanical performance, specimen reproducibility, and casting workability within the hand-pour open-cast format evaluated in the present study.

### Evaluation of PU foam as a cancellous bone analog

The PU foam used as the cancellous bone analog had a manufacturer-specified free-rise density of 27–31 kg/m³. The mold-constrained apparent density, measured gravimetrically from dumbbell specimens in the present study, was 280 ± 4 kg/m³ (0.280 g/cm³), approximately one order of magnitude higher than the free-rise specification, consistent with physical compression of the expanding cellular structure when expansion is resisted by the mold walls. This value lies at the lower boundary of the 0.16–0.64 g/cm³ (160–640 kg/m³) range typical of validated rigid PU cancellous bone analogs used in orthopedic simulation [10], and the modulus overlap therefore corresponds to the lower end of the trabecular density spectrum. The measured elastic modulus of 57.72 ± 7.69 MPa falls within the lower portion of the reported range for canine trabecular bone (approximately 50–14,000 MPa depending on anatomical site and apparent density [33, 34, 35]), a span that reflects the strong dependence of cancellous mechanical properties on apparent density [8]. This mechanical correspondence should be interpreted with caution because it is more consistent with low apparent density or osteoporotic-equivalent trabecular tissue than with normal canine cancellous bone. The foam was selected because of its commercial availability in Thailand, controllable expansion properties, and established use in orthopedic simulation [13, 14], and its elastic modulus is sufficiently distinct from all cortical composite groups to produce a mechanically differentiated bilayer construct, the primary functional requirement at this stage of model development.

### Development of a hybrid bilayer canine CBM

The mechanical profiles characterized in the present study provide the material basis for a proposed hybrid bilayer CBM combining a fiber-reinforced epoxy cortical shell with a PU foam cancellous core. The two components differ by more than one order of magnitude (23-fold) in elastic modulus, with E+3%RF exhibiting an elastic modulus of 1,333.06 ± 18.42 MPa and PU foam exhibiting an elastic modulus of 57.72 ± 7.69 MPa. This qualitative difference reproduces the structural hierarchy of natural bone, in which a stiff cortical envelope encloses a compliant trabecular interior [7, 8], although both values remain below the target ranges for canine cortical bone (7,500–21,000 MPa) and mid-range trabecular bone (approximately 100–800 MPa [33, 34, 35]), respectively. Exact mechanical equivalence to natural tissue is not required for effective procedural training; synthetic models with simplified mechanical properties have supported valid skill acquisition in veterinary surgical education [5, 6, 46]. This modulus differential is nevertheless a necessary condition for a bilayer surgical training model because it governs the tactile transition encountered when a drill or implant passes from cortical to cancellous tissue [9], although whether this magnitude is sufficient cannot be confirmed from tensile coupon data alone and requires functional testing. The low coefficient of variation for the elastic modulus of E+3%RF (1.4%) suggests that the cortical component can be fabricated with sufficient batch-to-batch consistency for standardized model production using a hand-pour process, although this inference requires confirmation in a three-dimensional mold geometry. Unlike monolithic synthetic bone constructs or human-focused commercial analogs [9, 10], a hand-pour epoxy-PU foam bilayer platform is, in principle, scalable to canine-specific mold geometries without specialized equipment, making it a candidate fabrication route for low-resource veterinary training settings. However, this scalability has not yet been demonstrated in an integrated construct.

### Cost-effectiveness of the proposed canine bone model

The estimated raw material cost for the proposed in-house canine femur model, comprising a 100 g E+3%RF cortical shell and a PU foam cancellous core, is approximately USD 1.90 per unit, representing approximately 7% of the USD 28.25 manufacturer’s suggested retail price of the equivalent Sawbones composite canine femur (Table 3[42]). The projected raw material saving per 10-model training set is USD 263 before international shipping and import costs. E (EP-089) and R (R804) were available in Thailand at USD 19.01/kg and USD 7.56/kg, respectively, and all materials required for fabrication are obtainable without specialized procurement. Cost-effective, locally fabricated training models are increasingly recognized as valuable tools for veterinary and surgical skills education, particularly in resource-limited settings [43–45]. Pink* et al.* [46] demonstrated a comparable hand-pour approach using PU foam coated with E fabricated from locally sourced materials in a lower-income setting, with drilling and K-wire performance acceptable for procedural training. The present study builds on that work by systematically characterizing the material mechanics and introducing fiber reinforcement to improve cortical stiffness, thereby providing a reproducible material baseline that informal fabrication approaches have lacked.

**Table 3:** Estimated raw material cost for the proposed in-house canine femur model fabricated using E+3%RF epoxy composite and PU foam compared with equivalent commercial composite bone models.

A. IN-HOUSE MODEL (E+3%RF + PU foam)		
Component	**Amount**	**Cost (USD)**
**Epoxy resin EP-089 (cortical shell)**	97 g	1.84
**Chopped E-glass fiber, 3 ** **wt** **% (cortical shell)**	3 g	0.05
**PU foam, 2-component (cancellous core)**	~0.5 mL	0.01
**Raw material cost per model**		~1.90
B. COMMERCIAL REFERENCE†		
Model (SKU‡)		**Price (USD)**
**Canine Femur, Foam Cortical Shell, Medium (2121)**		28.25
**Canine Femur, Short Oblique Fracture (2121-24)**		35.5
**Canine Femur, Long Oblique Fracture (2121-31)**		36.75
C. COST COMPARISON vs. SKU 2121		
**Absolute saving per model**		26.35
**In-house cost as % of commercial price**		~7%
**Saving per 10-model training set**		~263

Note: Material unit prices: epoxy resin EP-089, USD 19.01/kg; chopped E-glass fiber, USD 15.15/kg; PU foam, USD 20.84 per 2 L. Cancellous core volume estimated from medullary canal geometry (diameter 5 mm, length 15 cm; V ≈ 3.0 cm³); liquid PU volume rounded up to 0.5 mL. Cost does not include mould fabrication, ancillary equipment, or fabrication time. † Sawbones (Pacific Research Laboratories, Vashon, WA, USA); prices are US MSRP (sawbones.com, accessed June 2026), excluding international shipping and import duties. ‡ SKU = stock keeping unit.

### Limitations and future directions

Mechanical characterization was restricted to uniaxial tensile testing under quasi-static loading in accordance with ASTM D638, providing a reproducible basis for comparing material compositions but not a complete mechanical profile. Canine long bones are predominantly subjected to compression, bending, and torsion during physiological loading [47]; however, none of these loading modalities were evaluated in the present study. Fiber orientation in the hand-poured specimens was not characterized at the microstructural level. Random fiber distribution, the expected outcome of manual mixing and open casting, introduces anisotropy that cannot be resolved using tensile coupon data alone. Fatigue, cyclic, and torsional testing were not performed. Screw pull-out resistance, drilling behavior, plate fixation mechanics, and the effects of humidity exposure and sterilization on composite integrity also remain unquantified. A minimum of five specimens per group was used in accordance with ASTM D638. Although this satisfies the standard's minimum requirement for materials characterization, it represents the lower boundary of statistical power for detecting intergroup differences, and the findings should therefore be interpreted accordingly. The E+5%RF group was further reduced to n = 4 following pre-test grip failure of one specimen, thereby limiting confidence in both the mean value and the coefficient of variation at that concentration.

Three priorities emerge from these limitations. The immediate priority is functional validation of E+3%RF under loading conditions relevant to surgical training, specifically compressive testing, drilling resistance, and screw pull-out testing, because whether the remaining cortical stiffness gap represents a practical limitation depends on material behavior under drilling and implant loading rather than under uniaxial tensile loading alone. If functional testing confirms adequate performance, the hand-pour epoxy platform will be suitable for deployment without additional material reformulation. If functional testing does not confirm adequate performance, reducing the stiffness gap will require continuous or woven carbon fiber reinforcement or aligned fiber lay-up to improve fiber-matrix load transfer [15, 30, 31], because the short-fiber hand-pour format cannot achieve the 7.5–21 GPa elastic modulus of natural canine cortical bone [7] under its current design constraints. Optimization of the cancellous core toward the 0.16–0.32 g/cm³ apparent density range of validated rigid PU cancellous bone analogs, and toward breed- and site-specific mechanical targets, is most practically achieved using a three-dimensional printed lattice geometry, which would decouple apparent density from mold constraint and permit direct alignment with published canine trabecular bone properties [33–35]. Once a construct meeting the functional requirements for surgical training has been fabricated and validated, a blinded comparative study evaluating fracture stabilization performance using synthetic and cadaveric specimens, assessed by expert raters, would determine whether the proposed platform provides training outcomes sufficient to support its recommendation as a cadaver-reduction tool in veterinary surgical curricula.

## CONCLUSION

This study systematically evaluated hand-pour fabricated fiber-reinforced E and R composites together with PU foam to identify an affordable material combination for canine CBM fabrication. Among the formulations evaluated, E+3%RF provided the most favorable overall balance of mechanical performance, manufacturing reproducibility, and casting feasibility. Fiber reinforcement significantly increased the elastic modulus and yield strength of epoxy composites while reducing ultimate stress and ultimate strain, producing mechanical behavior that more closely approximated canine cortical bone than unreinforced E. In contrast, R showed no significant improvement in elastic modulus across the tested fiber weight fractions, most likely because cure shrinkage limited efficient fiber-matrix load transfer. The PU foam exhibited an apparent density and elastic modulus consistent with the lower end of the reported canine trabecular bone spectrum, providing a mechanically distinct cancellous component suitable for incorporation into a bilayer construct.

From a practical perspective, the proposed E+3%RF cortical shell combined with a PU foam cancellous core offers a simple, low-cost, and locally manufacturable alternative for developing canine orthopedic training models. The estimated raw material cost of approximately USD 1.90 per model, representing about 7% of the cost of a comparable commercial model, demonstrates the potential to substantially reduce training expenses while improving accessibility for veterinary institutions, particularly in resource-limited settings. The low coefficient of variation observed for E+3%RF further supports its suitability for standardized batch production using a hand-pour fabrication process.

A major strength of this study is the systematic comparison of Eand R systems across multiple fiber weight fractions using standardized mechanical testing, thereby providing the first evidence-based optimization of fiber reinforcement specifically for canine orthopedic training model fabrication. However, the study was limited to quasi-static uniaxial tensile characterization, and the proposed composite system has not yet been validated under clinically relevant loading conditions such as compression, bending, torsion, drilling, or screw fixation. In addition, the mechanical performance of the integrated bilayer construct remains to be established.

Future studies should evaluate the complete hybrid CBM under functional orthopedic procedures, optimize the cancellous core architecture to better reproduce canine trabecular bone, investigate alternative reinforcement strategies to further improve cortical stiffness, and compare training outcomes between the proposed synthetic model and cadaveric specimens. Such investigations will determine whether this affordable composite platform can serve as a reliable substitute for cadaveric bone in veterinary surgical education and contribute to implementation of the 3Rs principle through reduced dependence on animal-derived teaching materials.

## DATA AVAILABILITY

The datasets generated and/or analyzed during the current study are available from the corresponding author upon reasonable request.

## GENERATIVE AI DECLARATION

The authors declare that generative artificial intelligence (AI) tools were used solely to improve language, grammar, and readability during manuscript preparation. All scientific content, data analysis, interpretation of results, and conclusions were developed and verified by the authors. The authors take full responsibility for the accuracy, integrity, and originality of the work presented, and no AI tool was listed as an author.

## AUTHORS’ CONTRIBUTIONS

SS and NC: Conceived and designed the study. SS and SV: Conducted the literature review, fabricated the specimens, collected the data, and drafted the original manuscript. NC: Performed the mechanical testing. SS: Performed the statistical analysis and data visualization. SS, SV, AC, and NC: Reviewed and critically revised the manuscript. All authors have read and approved the final manuscript.
